# Marine-Sourced Anti-Cancer and Cancer Pain Control Agents in Clinical and Late Preclinical Development ^†^

**DOI:** 10.3390/md12010255

**Published:** 2014-01-14

**Authors:** David J. Newman, Gordon M. Cragg

**Affiliations:** Natural Products Branch, Developmental Therapeutics Program, Division of Cancer Treatment and Diagnosis, Frederick National Laboratory, P.O. Box B, Frederick, MD 21702, USA; E-Mail: gmcragg@verizon.net

**Keywords:** Antibody Drug Conjugates (ADCs), marine antitumor agents, clinical trials, approved antitumor agents

## Abstract

The marine habitat has produced a significant number of very potent marine-derived agents that have the potential to inhibit the growth of human tumor cells *in vitro* and, in a number of cases, in both *in vivo* murine models and in humans. Although many agents have entered clinical trials in cancer, to date, only Cytarabine, Yondelis^®^ (ET743), Eribulin (a synthetic derivative based on the structure of halichondrin B), and the dolastatin 10 derivative, monomethylauristatin E (MMAE or vedotin) as a warhead, have been approved for use in humans (Adcetris^®^). In this review, we show the compounds derived from marine sources that are currently in clinical trials against cancer. We have included brief discussions of the approved agents, where they are in trials to extend their initial approved activity (a common practice once an agent is approved), and have also included an extensive discussion of the use of auristatin derivatives as warheads, plus an area that has rarely been covered, the use of marine-derived agents to ameliorate the pain from cancers in humans, and to act as an adjuvant in immunological therapies.

## 1. Introduction

Rather than discuss the agents that are currently in use from marine sourced organisms, which will be covered in another review in this journal, we will discuss agents that are from marine or marine-derived sources that are either in clinical trials, or are in advanced preclinical status. Obviously we will not be covering all such agents, as some are known only by a code number without any other information being available, whilst others are in “preclinical status” according to the authors of a paper or communication, but in truth, most of these are simply reports of some *in vitro* activity against cell lines or have some preliminary data on *in vivo* activity in rodents.

We will also avoid using the source organism as the method of classification as it is now becoming quite evident that the majority of compounds reported from the marine environment are in fact produced by, or in concert with, single-celled organisms ranging from protists (frequently dinoflagellates) to bacteria, including a very significant number of as yet uncultured organisms.

We will mention some of the materials that have been approved for use in one or more countries that are in fact in clinical trials in others, or are now being used in conjunction with other drug moieties as these are very common occurrences with antitumor agents once they are approved. For example, although not a marine-derived agent, taxol^®^ is still in clinical trials, usually as part of a multi-drug regimen more than 20 years after it was approved for use by the US Food and Drug Administration (FDA) for treatment of refractory ovarian cancer.

We have organized this review in a manner that is the reverse to what most authors would do, in that we will commence with agents that have been approved but are still in clinical trials, followed by agents in stages of clinical development (nominally Phase I to III), rather than start with preclinical agents and work forwards.

Since a number of the agents that are in clinical trials are very close relatives to approved materials, we have elected to group these agents after the “approved parent”, so that the similarities and differences can be more easily seen, thus giving the full “chemical lineage” in certain cases below. In addition, we have elected to commence with compounds from marine sources that could be considered as “adjuvant therapies” though, with one exception, not in the immunological sense.

## 2. Treatment of Pain Associated with Cancer

### 2.1. Tetrodotoxin (Tectin ^®^, Phase III; Figure 1, **1**)

One of the most unusual agents at this stage is a very well known “marine toxin”, the highly substituted guanidine-derivative, tetrodotoxin (**1**) [1,2,3]. Although this is not a formal anti-tumor agent, it is in fact in Phase III trials as an agent (Tectin^®^) against inadequately controlled pain related to cancer by WEX Pharmaceuticals in the USA, together with a Phase II trial under the same company, again in the USA, against the neuropathic pain resulting from chemotherapy-induced peripheral neuropathy. Although there was debate in years gone by over the actual source of this agent, there is now little doubt that it is produced by a commensal microbe, though which one(s) is still open for debate [4]. The synthesis of the compound and other derivatives has been published from a variety of chemists with an excellent recent review by Nishikawa and Isobe giving the highlights of their methodologies and covering some of the early history of this class of toxins [5].

**Figure 1:**
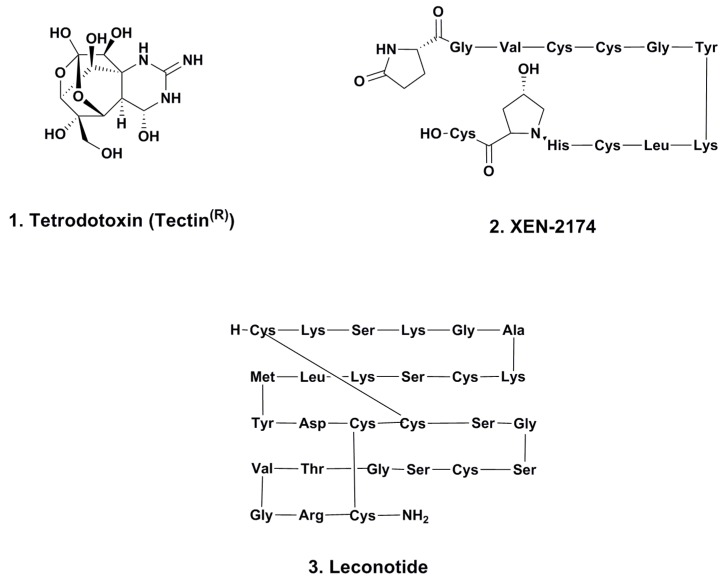
Pain control agents

### 2.2. XEN-2174 (Phase II; Figure 1, **2**)

This compound, a very slight modification of the naturally occurring χ-conotoxin MrIA, was originally isolated from *C. marmoreus* and then optimized by medicinal chemistry [6]. Unlike the other conotoxins either approved or in various levels of testing, this particular agent is a modified 13-residue peptide and is a noncompetitive inhibitor of the neuronal norepinephrine transporter (NET) [7].

### 2.3. Leconotide (AM-336, ω-Conotoxin CVID; Phase I; Figure 1, **3**)

This molecule, a 27 residue peptide with three internal CYS-CYS bonds, is similar to the well-known pain treatment ziconotide, and is currently in Phase I trials sponsored by Relevare Pharmaceuticals (previous name was CNSBio) for treatment of pain related to cancer. It is a calcium channel blocker and was originally identified by researchers at the University of Queensland. Although initial experiments used the intrathecal route (as with ziconotide) [8], the current protocol uses systemic administration [9].

### 2.4. Immunological Use of Keyhole Limpet Hemocyanin (KLH; Phase I–III)

KLH has been used for many years as a classical immunoadjuvant, and had been approved in countries from Austria to South Korea, mainly for treatment of bladder cancer [10]. Two recent publications gave results from Phase III trials, the first being in metastatic breast cancer where it did not demonstrate any increase in median life span [11], but in the other Phase III trial in bladder cancer, using mitomycin as a comparative agent, there were indications that KLH had a positive effect on disease progression [12]. Currently, the ClinicalTrials.gov web site [13] lists Phase III (NCT01480479) and Phase II (NCT01498328) trials using KLH in its adjuvant status against relapsed glioblastoma, and Phase I trials in conjunction with KLH as part of a vaccine against high risk neuroblastoma (NCT00911560) and fallopian tube, epithelial ovarian and peritoneal cancers in patients following a first remission (NCT01248273).

## 3. Approved Marine-Derived Antitumor Agents Still in Clinical Trials (and Close Chemical Relatives)

### 3.1. Cytarabine (Phases I to IV; Figure 2, **4**)

As mentioned in a news interview in the early 1990s and then formally in a review by the authors in 2000 [14] this agent, though not found in a marine environment as “Ara-C” can trace its chemical lineage back to the discovery of bioactive nucleosides that contained arabinose rather than ribose or deoxyribose. Though we were not the first to recognize the importance of such substitutions, as Suckling [15] in a review in 1991 reported on the chemistry involved in the syntheses of these and other such arabinose-linked nucleosides with common or uncommon bases, we were perhaps the first to formally link the discoveries of the marine-sourced natural arabinoses by the Bergmann group to the “design” of this agent [16,17,18]. So Ara-C can be legitimately considered to be a marine-derived agent, since without the arabinose, it would simply have been a normal component of nucleic acids.

Even today, there are 840 trials listed in the NIH (National Institutes of Health, Bethesda, MD, USA) clinical trials database (ClinicalTrials.gov), with 240 of them being open studies that are recruiting, covering a large number of cancers and ranging from Phase IV down to Phase I. In the corresponding European database, 43 clinical trials covering the same phases, but with some overlap, are listed. As with other well-known approved drugs, it is still in use, more than 40 years after its initial approval, with an interesting recent paper questioning the use of high dose cytarabine therapy during remission in adults of acute myeloid leukemia [19].

### 3.2. ET743 (Trabectedin; Yondelis^®^; Phases I to III; Figure 2, **5**)

This compound may well be considered the “poster child” for marine-derived antitumor agents, as it is currently the only molecule in use as an antitumor agent that is identical to one of the compounds originally isolated from *E. turbinata*. The stories around the discovery and development of this compound using materials from in-sea and on land aquaculture, followed by the semi-synthesis from a precursor molecule from a marine microorganism, cyanosafracin B, have been told by many authors over the years. These ranged from the initial reports of bioactivity in this organism in 1970 by Sigel *et al.* [20], the initial identification of the series by Holt in his PhD thesis in 1986 [21], to the simultaneous publications from the laboratories of Rinehart at the University of Illinois (Urbana Champaign, IL, USA) [22], and Wright at Harbor Branch Oceanographic Institution (Fort Pierce, FL, USA) [23] in 1990 of the structure of ET743. This work was followed with the thorough discussion given by the investigators at PharmaMar (Madrid, Spain) in 2009 [24], demonstrating the power of both semi-synthesis and optimization of processes to obtain active drug principles. The molecule was approved in the EU (European Union) in 2007 for treatment of advanced soft tissue sarcoma and in some of the EU countries for treatment of recurrent platinum-sensitive ovarian cancer when coupled to liposomal doxorubicin in 2009, but the corresponding U.S. FDA (Food and Drug Administration) application was withdrawn.

**Figure 2:**
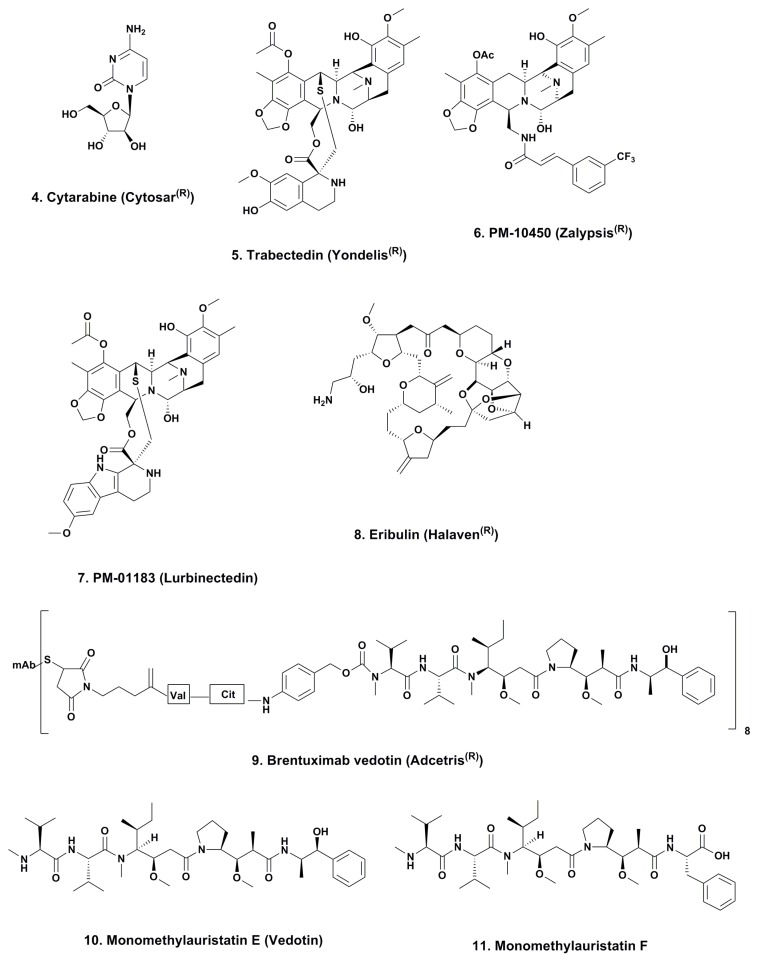
Approved marine-derived drugs and close analogues in clinical trials.

The commonalities and differences in the pharmacological response of trabectedin and its close relatives, Zalypsis^®^ and lurbinectedin (*vide infra*) have been discussed recently with respect to their experimental effect upon the Fanconi anemia pathway. Martinez *et al.* [25] demonstrated that these three agents inhibited the Fanconi anemia pathway in the cell lines tested, increasing their sensitivity to mitomycin C, in contrast to mitomycin C which always activated that pathway in the same cell lines. The authors suggested that as a result of these findings these three agents might be useful clinically in “Fanconizing” cancer cells in order to gain sensitivity against other anti-tumor drugs. In another paper the same year, Romano *et al.* [26] reported that in *in vitro* and *in vivo* models, no relationship was found between the *in vitro* cytotoxic potency and *in vivo* antitumor activity in syngeneic mouse models, suggesting that there might well be a host response in these models. In addition, the pharmacokinetics differ, even between the quite similar trabectedin and lurbinectedin in humans, and as expected due to the differences in structure, Zalypsis^®^ has been shown to differ in pharmacokinetics in humans [27].

As of the end of October 2013, there were 15 studies found for ET743 in the NIH clinical trials database, 12 at Phase II and three at Phase I, all being listed as completed, with cancer types covering breast, prostate, soft tissue sarcoma and osteosarcoma, plus general carcinomas. Searching the corresponding EU Clinical Trials Register, 19 trials were listed ranging from 2005 to 2013 with three being Phase III trials not found on the NIH site. These were two trials against refractory ovarian cancer with liposomal doxorubicin, and the third was for patients with translocation-related sarcomas. Again these listings demonstrates that once a compound has been approved for treatment of one type of cancer, it will be placed in clinical trials for many others, either individually or as part of a new drug regimen.

A discussion of the probabilities of ET743 and its congeners being produced by as yet uncultured microbes associated with the source tunicate was recently published by Giddings and Newman [28] which should be consulted for further details.

#### 3.2.1. PM-10450 (Zalypsis^®^; Phases I–II; Figure 2, **6**)

This compound, another variation on the basic structure of the dimeric isoquinoline alkaloids, was derived from the structure of jorumycin, a compound isolated from the skin and mucin of the nudibranch *Joruna funebris* [29], and renieramycin J from a species of the marine sponge, *Netropsia*. Zalypsis^®^ was synthesized by workers at PharmaMar (Madrid, Spain) using methodologies related to the ET743 synthesis from safracin B [30]. The initial report of the molecular pharmacology of this agent was described by Leal *et al.* in 2009 [31] and even though it has a close resemblance to ET743, it does not activate the DNA damage checkpoint response.

Currently both the NIH and EU clinical trials sites show three clinical trials at Phase II/I levels with one in Spain showing as still continuing. There are eleven reports to date in the literature with recent results from Phase I studies being reported in 2012 from work in the UK [27]. These were then followed by further reports in 2013, where objective responses, mainly stable disease, were seen in a small number of patients [32,33]. In contrast, also in 2013, a report was published demonstrating a lack of response and termination of the Phase II trial of this compound in a heavily pretreated population with advanced and/or metastatic endometrial or cervical cancers [34].

#### 3.2.2. Lurbinectedin (PM-01183; Phases I–II; Figure 2, **7**)

This compound is another variation on the basic structure of the dimeric isoquinoline alkaloids, but has a tetrahydro-β-carboline moiety instead of the tetrahydroisoquinoline present in ring C, and binds in the DNA minor groove [35]. The compound was shown to have different pharmacokinetics in patients and also like trabectedin, to attenuate nuclear excision repair (NER). It also demonstrated synergy with platinum-based agents *in vitro* thus suggesting a possible treatment regimen since it also demonstrated activity against platinum-resistant cell lines [36]. Two Phase II clinical trials with lurbinectedin are shown on the NIH clinical trials site, one recruiting and one approved but not yet recruiting, with two Phase I trials recruiting and one approved but not yet recruiting. On the European clinical trials site, one Phase II trial corresponding to the” not yet recruiting trial” listed on the NIH site in the USA, is on-going in Spain, and the other is a two year old trial against metastatic pancreatic cancer in Spain and the UK.

### 3.3. Eribulin (Halaven^®^; Phases I–IV; Figure 2, **8**)

The story of the discovery of this compound (a totally synthetic variation on halichondrin B has been given in a variety of formats over the years, from the chapter by the Eisai scientists in Woburn, MA that showed the progression from the synthesis of halichondrin B to the initial synthesis of eribulin [37], to two recent papers on the industrial methodologies that enabled the production of this molecule, certainly the most complex synthetic drug to date [38,39].

As with the other approved compounds mentioned earlier, Halaven^®^ is currently shown as being in 28 trials that are recruiting patients with 27 being Phase I or II or I/II. The one Phase III trial is a physician’s choice model with Halaven^®^ being one of the three drugs to choose from. In addition, of the other 43 trials shown, 21 are active but not recruiting with the majority being at Phase I or II, though two are at Phase III and one (Phase IV) is a post market surveillance. One trial in the list was terminated with no reason given. In addition, a new liposomal formulation of eribulin mesilate is currently in a Phase I clinical trial (NCT01945710) in the United Kingdom under the auspices of Eisai.

The geographic areas of these trials effectively cover the world, but the majority are either in the USA or Europe. Summation of the figures on the map in the Clinicaltrials web site always gives a higher figure as a significant number of trials cross geographic boundaries within the one trial.

### 3.4. Brentuximab Vedotin (Adcetris^®^; Phases 0 to IV; Figure 2, **9**)

This immunoconjugate with a “warhead” derived from dolastatin 10, monomethylauristatin E (vedotin; Figure 2, **10**), a secondary metabolite from a *Symploca* species of cyanophyte, was approved in the USA in 2011 for treatment of CD30 positive lymphoproliferative disorders such as Hodgkin’s lymphoma. This combination was the second immunoglobulin-warhead combination to be approved for leukemias following the initial approval of Mylotarg^®^ by the FDA in 2000.

Subsequently Mylotarg^®^ was withdrawn in the USA in 2010 due to concerns about the productʼs safety that were raised by a confirmatory study conducted after approval, as patients on the preparation and also receiving chemotherapy had a higher death rate and no objective increase in life when compared to a group using just chemotherapy. This combination is still in use in other countries. A relationship to marine sources for the “warhead endiyne molecule” was established when investigators at the Scripps Oceanographic Institution (La Jolla, CA, USA) showed the presence of endiyne cryptic clusters in marine bacteria of the genus *Salinospora* [40].

Adcetris^®^ is the product of extensive work by Seattle Genetics (Seattle, WA, USA), first in optimizing the vedotin warhead (**10**) and then developing the linkers that couple the antibody to the compound [41]. Some of these, discussed later, are designed to release the warhead (vedotin) by simple hydrolysis of a linker bond, whereas others require the enzymatic digestion of the antibody, releasing the warhead plus appendages. It was approved by the FDA in 2011 and subsequently approval was given in the EU late in 2012 and launched in the UK in early 2013, all for CD30 positive leukemias. Full explanations of the methodologies used and the utility of this agent against a variety of lymphomas have been published in the last three years and should be consulted by the interested reader [42,43,44,45]. In addition, a recent report from Takeda (Osaka, Japan) shows the strategy that this company is adopting, including the further development of this agent [46].

Currently, this agent is in 37 trials mainly in the USA from Phase 0 to Phase IV where the latter trial is listed as recruiting on the NIH clinical trials site. Six more are listed in the EU clinical trials site covering Phase II to Phase IV.

#### 3.4.1. Glembatumumab Vedotin (Phase II)

This is monomethylauristatin E (MMAE) linked to a fully human monoclonal antibody CR011 (an anti-CG56972) via a stable valine-citrulline dipeptide linker. It was targeted against patients with unresectable melanomas at stage III or IV who have failed one cytotoxic chemotherapy regimen and has expanded to include metastatic breast cancer as well. The combination has a variety of names during its early days including CDX-011, CR-011 and CR011-vcMMAE, so searching for data can be a trifle challenging.

The initial report of the use of this combination was given by investigators from CuraGen in 2006 [47], followed by a report of xenograft activity in 2007 from the same group [48]. The value of the monoclonal’s target in triple negative breast cancer was described in 2010 by Rose *et al.* [49], with the corresponding details in melanoma described in 2012 by a group from the People’s Republic of China [50]. Currently three completed studies at the Phase I/II levels are reported in the NIH clinical trials database with one preliminary report of clinical activity in breast cancer patients [51].

#### 3.4.2. ABT-414 (Phase I–II)

This is an antibody-drug conjugate (ADC) linking the anti-Epidermal Growth Factor Receptor (EGFR) antibody ABT-806 to another variation on auristatin; in this case, monomethylaurisatin F (Figure 2, **11**) is used in place of the “E” variant. The ADC was designed to bind to a unique epitope of EGFR that is usually not accessible when EGFR is expressed at physiological levels. However, the ADC binds when tumors express EGFRde2-7 (EGFRvIII) and in other tumors with amplified EGFR or excessive EGFR activation under “normal wild-type conditions” [52].

Abbvie (North Chicago, IL, USA), which is the renamed Abbott Pharmaceutical Division, recently instituted two human clinical trials as trials in mice using human wild-type EGFR-overexpressing tumors gave complete regressions and “cures” [52]. Phase I studies where patients must have a solid tumor type likely to over-express (EGFR), are underway evaluating the safety, pharmacokinetics and efficacy of ABT-414, with a Phase IIa expansion (NCT 01741727) at the maximum tolerated dose (MTD) where patients, accepted by invitation only, must have squamous cell Non-Small Cell Lung Cancer (NSCLC). The other trial at the Phase I level (NCT01800695) is a study evaluating the safety and pharmacokinetics of ABT-414 in subjects with glioblastoma multiforme in combination with radiation plus temozolomide or temozolomide alone; the study is currently recruiting patients with this particular disease.

#### 3.4.3. PSMA-ADC (Phase II)

This ADC is a fully human monoclonal antibody against prostate specific antigen that is coupled via the valine-citrulline dipeptide linker to mono-methylauristatin E (MMAE) and was designed to undergo release via proteolysis by human cathepsin B. The initial report demonstrating activity in prostate cancer cells and in xenografts was published in 2006 [53]. This was followed in 2011 by a report showing expanded activity against androgen sensitive and insensitive cell lines in xenografts [54]. 

Since there are now reports of the PSMA antigen being present in glioblastoma multiforme, this ADC is in a Phase II trial currently recruiting patients with this specific cancer, in addition to the Phase I and II trials against prostate cancer. All three trials are listed as current in the NIH clinical trials database, but no trials are shown in the EU database at this time.

#### 3.4.4. DCDT-2980S (Phase II)

This ADC from Genentech (Roche, San Francisco, CA, USA) is a humanized IgG1 antibody directed against the CD22 epitope linked to sulfhydryl groups on the antibody via a maleimide derivative. This derivative is the same as that used in Adcetris^®^, releasing monomethylauristatin E on protease cleavage. Since the CD22 epitope is not expressed in rodents, trials for safety were performed in cynomolgus monkeys and demonstrated adequate safety in primates plus efficacy in xenografts [55]. Currently there is one Phase II trial recruiting and one active trial in the NIH clinical database and no records in the EU equivalent. These trials are in leukemias, not solid tumors.

#### 3.4.5. DCDS-4501A (Phase II)

This is also from Genentech/Roche, and is an ADC with monomethylauristatin E linked to an anti-CD79b monoclonal. It is currently in the same Phase II trial as DCDT-2980S as an alternative treatment against follicular B cell lymphoma, and is also in an ongoing Phase I trial against various lymphomas in a dose escalation study. As with the earlier Roche agent (Section 3.4.4), no trials are listed in the EU database.

#### 3.4.6. Enfortumab vedotin (Phase I)

This combination, a fully human IgG1k antibody linked to monomethyl auristatin E via a cleavable valine-citrulline linker is also known under the code names AGS-22MSE and AGS-22ME and is currently undergoing Phase I evaluation under the aegis of Agensys (Ashburn, VA, USA), Seattle Genetics and Astellas Pharma (Tokyo, Japan). It should be pointed out that Agensys is a subsidiary of Astellas Pharma and the philosophy behind the approach from their standpoint was reported by Yanagita and Takenaka in 2012 [56]. The only record at the moment of the initial development of this agent is in an abstract of the 2011 AACR Meeting in Orlando, Florida [57].

#### 3.4.7. Vorsetuzumab Mafdotin (SGN-75; Phase I)

This ADC, from Seattle Genetics, has monomethylauristatin F linked to the humanized anti-CD70 monoclonal antibody 1F6 through a maleimidocaproyl linker that is non-cleavable, so the release has to rely upon invagination and then proteolytic digestion [58]. This ADC is currently being evaluated in Phase I trials against relapsed and refractory non-Hodgkin’s lymphoma and also metastatic renal cancer where the cancers express the CD70 epitope. Currently the NIH database shows one completed Phase I trial and one recruiting for renal cell carcinoma, but no trials in the EU database. There are reports in the conference literature on some of the findings, but as yet, no peer-reviewed reports.

#### 3.4.8. SGN-19A (SGN-CD19A; Phase I)

This is another Seattle Genetics ADC where a humanized anti-CD19 antibody is linked to monomethylauristatin F through a maleimidocaproyl-valine-citrulline linker. Currently there are two Phase I trials in the NIH database at the Phase I level that are actively recruiting for trials in lymphomas including Burkitt’s lymphoma. One presentation at the 2013 AACR meeting is the only published report of progress at the moment [59].

#### 3.4.9. BAY 79-4620 (3ee9/MMAE; Phase I)

This ADC is monomethylauristatin E linked to an antibody against the human carbonic anhydrase IX, and was made using the Seattle Genetics techniques as described for the anti-CD30 ADC (now known as Adcetris^®^) by Francisco *et al.* in 2003 [60]. Two Phase I trials are listed in the NIH database with one showing completed (determination of MTD in patients with advanced solid tumors) but the other, again an MTD-based study, was terminated for safety reasons. The differences between the two trials from the database descriptions appeared to be the frequency of treatment, three weeks in the completed trial *versus* two weeks in the terminated one. No data in the EU database.

#### 3.4.10. AGS-16C3F (AGS-16M8F; Phase I)

This ADC is a fully human IgG2k monoclonal antibody directed against the AGS-16 antigen and conjugated to monomethylauristatin F (MMAF) via a noncleavable maleimido-caproyl linker. This particular ADC is directed against renal and liver carcinomas as a result of the AGS-16 antigen. Details of the initial discovery and results of cell line and xenograft testing were presented at the 2010 Genitourinary Cancers Symposium by Gudas *et al.* [61]. Currently there is one Phase I trial against renal cancer recruiting according to the NIH database, and one completed Phase I looking at the safety of dose escalation.

#### 3.4.11. DMUC-5754A (RG-7458; Phase I)

This ADC is a monoclonal antibody against the epitope MUC16 linked to monomethylauristatin E and it is targeted against ovarian carcinomas, but no further details other than a report in an abstract at the 2013 AACR meeting [62], are available at the present time. Currently the NIH web site shows that Genentech is recruiting patients for a Phase I trial against both ovarian and pancreatic cancer.

#### 3.4.12. DNIB-0600A (RG-7599; Phase I)

This ADC is a humanized IgG1 monoclonal antibody directed against the NaPi2b epitope linked to monomethylauristatin E. No information as to the method of linkage is available and the only report is in an abstract at the 2013 ASCO meeting [63]. Phase I trials against non-small cell lung cancer and platinum resistant ovarian cancer are actively recruiting patients according to the NIH database.

#### 3.4.13. A1-mcMMAF (PF-06263507; Phase I)

This ADC is monomethylauristatin F linked via a maleimidocaproyl linker to a humanized monoclonal antibody directed against the 5T4 tumor antigen. The ADC was prepared using the basic techniques described by Doronina *et al.* [41], and demonstrated potent antitumor activity in *in vivo* xenograft models and exhibited no overt toxicities when delivered to cynomolgus monkeys [64]. Currently Pfizer is recruiting patients for a Phase I trial against advanced solid tumors according to the NIH database.

#### 3.4.14. DMOT-4039A (Phase I)

This ADC is a monoclonal antibody identified as MMOT-0530A that was raised against an un-named antigen that is overexpressed in pancreatic and ovarian cancer, conjugated to monomethylauristatin E. Currently the ADC is in two Phase I clinical trials with one in the USA and The Netherlands recruiting patients with unresectable pancreatic or platinum-resistant cancers (NCT01469793), whilst the other one (NCT01832116), also directed against the same cancers and actively recruiting patients in The Netherlands, is designed to use PET imaging using ^89^Zr-linked to the MMOT antibody as the imaging agent, followed by use of the ADC thus permitting an assessment of the imaging and the subsequent response to therapy.

#### 3.4.15. RG-7600 (Phase I)

This ADC, which from the comments on the Genentech web site [65], is in Phase I studies against ovarian cancers, does not have any other information in the literature. However, since the web site states that it is in collaboration with Seattle Genetics, the warhead may be an auristatin derivative. No details as to current trials can be found in the NIH database.

#### 3.4.16. DEDN-6526A (RG-7636; Phase I)

As with RG-7600, the only information is from the Genentech web site where this ADC is listed as being in Phase I against unresectable melanoma. Since Seattle Genetics is also involved, the warhead may be an auristatin derivative, and the antibody may well be directed against endothelin ETB receptors. One trial that is recruiting patients is shown in the NIH database (NCT01522664).

#### 3.4.17. DSTP-3086S (RG-7450; thio-antiSTEAP1-MC-vc-PAB-MMAE; Phase I)

This is another of Genentech (Roche) ADCs using, in this case, an antibody against a humanized anti-STEAP1 IgG1 antibody modified via determination/modification of reactive thiols according to the patent application by Bhakta and Junutula [66], and coupled to monomethylauristatin E. The antibody is directed against the six-transmembrane epithelial antigen of prostate 1, hence the STEAP1 acronym, and was evaluated as both the basic ADC with monomethylauristatin E and the thio-modified antibody, with the decision being to go with the thio modified version from the PK determinations [67,68]. Currently there is one Phase I study recruiting patients with metastatic castration-resistant prostate cancer (NCT01283373) with a preliminary report showing some clinical responses given at the 2013 ASCO Meeting [69].

#### 3.4.18. MLN-0264 (Phase I)

This is an ADC composed of a fully human monoclonal IgG antibody (5F9) directed against guanylyl cyclase C (GCC) and is conjugated to monomethylauristatin E via a cleavable linker (licenced from Seattle Genetics). The antibody is directed against gastrointestinal tumors that express GCC. This is a first in class drug candidate with the initial report of the rationale being given in November 2012 at the 14th EORTC-NCI-AACR meeting in Dublin, Ireland [70]. A report at the next conference in the series was given in 2013, demonstrating activity both alone and in conjunction with gemcitabine in xenograft models of pancreatic cancer [71]. The compound is in a Phase I trial (NCT01577758) and is currently recruiting patients with GI cancers expressing the required antigen.

#### 3.4.19. RG-7598 (Phase I)

As with RG-7600 (Section 3.4.15), the only information is from the Genentech web site where this ADC is listed as being in Phase I against multiple myeloma. Since Seattle Genetics is also involved, the warhead is probably an auristatin derivative. No trials can be found in the NIH database as of early November 2013.

#### 3.4.20. SGN-LIV1A (Phase I)

This ADC is being developed by Seattle Genetics and is an anti-LIV-1 humanized monoclonal antibody linked to monomethylauristatin E. The LIV-1 epitope is also known as SLC39A6 or ZIP6, and is a member of the zinc transporter family. It was first identified as an estrogen-inducible gene in breast cancer derived cell lines. It is a downstream target of STAT3, and promotes the epithelial to mesenchymal transition important in the malignant progression of breast cancer to the metastatic form. It is expressed in subtypes of metastatic breast cancers (ER+/HER2−; HER2+ and triple negative). However, in healthy human tissues, its expression is limited to four hormonally-regulated organs. Both *in vitro* and *in vivo* models demonstrated significant delay of tumor growth on treatment with the ADC [72]. This ADC is in a Phase I study that is currently recruiting patients (NCT01969643) with metastatic breast cancer to determine safety and any antitumor activity during the trial.

#### 3.4.21. AGS-15E (AGS-15ME; Phase I)

This is an ADC with the fully human IgG2 monoclonal antibody (AGS15) whose target is SLITRK6, conjugated to monomethylauristatin E (MMAE) through the maleimidocaproyl-valine-citrulline linker from Seattle Genetics. The target of this antibody, SLITRK6, is a member of the SLITRK family of neuronal transmembrane proteins, and was discovered by Agensys using suppressive subtractive hybridization on biopsies from bladder cancer patients [73].

Immunohistochemical (IHC) analysis of SLITRK6 expression was evaluated in various human cancers including bladder, using a SLITRK6-specific antibody M15-68(2)22. This mouse monoclonal antibody demonstrated that 90% of 452 human bladder transitional cell carcinomas from *in situ*, advanced primary and metastatic tumors express this epitope. In addition, the same expression was seen in some lung, breast and glioblastomas as well. In normal tissues, expression is significantly restricted [73]. Currently a Phase I trial (NCT01963052) is actively recruiting patients with metastatic urothelial cancer.

### 3.5. Preclinical Auristatin-Linked ADCs

The following ADCs are currently in advanced preclinical trials but the current information that is available is minimal.

#### 3.5.1. CDX-014 (CR-014-vcMMAE)

From the code name, this is a valine-citrulline-linked monomethylauristatin E linked to a fully human anti-TIM1 monoclonal antibody CR-014. This antibody was developed to selectively target TIM 1 (Kd = 2.7 nM), a type I transmembrane protein expressed on the surface of ovarian and renal carcinoma cells with poor expression in normal tissues.

#### 3.5.2. HuMax-CD74

This auristatin-linked ADC in preclinical development uses HuMax-CD74, an antibody that targets HLA class II histocompatibility antigen gamma chain (CD74). This epitope is expressed in a wide range of hematological malignancies and solid tumors, and is being developed by a collaboration between Genmab (city, state, country) and Seattle Genetics [74].

#### 3.5.3. HuMab-TF-011-vcMMAE (HuMax-TF-ADC; TF-011-MMAE IND Filed)

TF-011-vcMMAE is an ADC under development for the treatment of solid tumors. It is composed of a human tissue factor (TF) specific antibody (TF-011), linked to a protease cleavable valine-citrulline (vc) linker and monomethylauristatin E (MMAE). TF is aberrantly expressed in many solid tumors, and its expression has been associated with poor prognosis [75].

## 4. Other Marine-Derived Compounds in Clinical Trials against Cancer (Phases I–III)

### 4.1. Aplidine (Ptilidepsin, Aplidin^®^; Phase II–III; Figure 3, **12**)

This compound is currently the only non-approved marine-derived agent in Phase III clinical trials for cancer. Its history is quite convoluted as it was originally identified from an extract of the Mediterranean tunicate *Aplidium albicans.* It was first reported in a patent from the Rinehart laboratory at the University of Illinois in Champaign-Urbana during the time that that laboratory was working on the synthesis of didemnin B (the first direct from the sea compound to go into human clinical trials against cancer) [76]. Subsequently, with the withdrawal of didemnin B due to toxicity and immunosuppressive effects which may have been exacerbated by the then current drug regimens (a bolus at the MTD), PharmaMar began developing aplidine.

**Figure 3:**
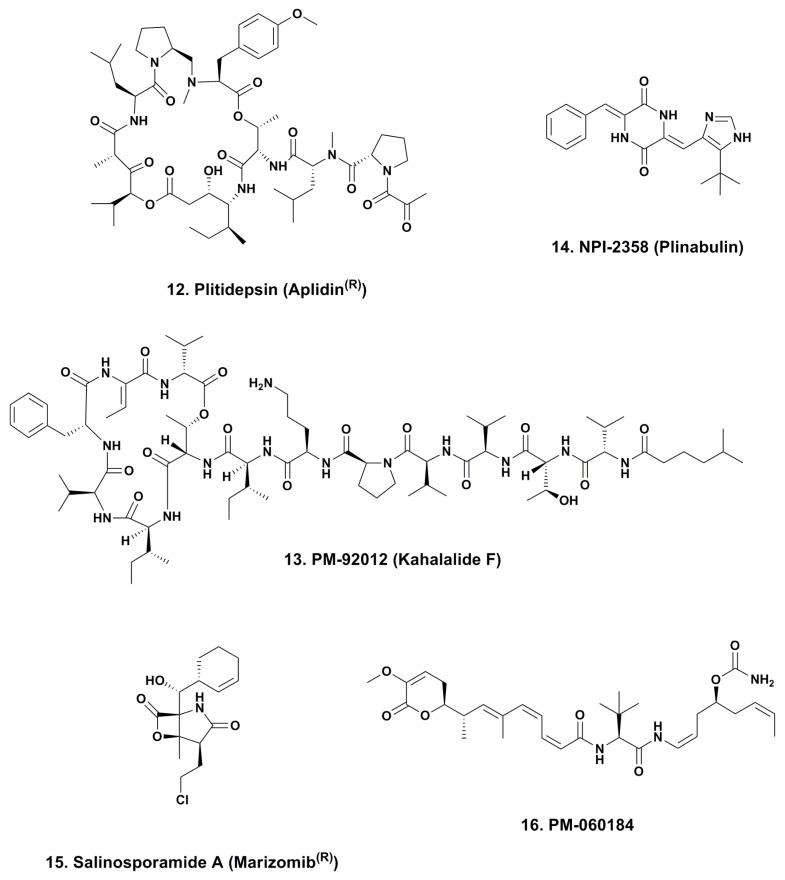
Other marine-derived compounds in phase I–III trials.

This time the agent was made by total synthesis using data from the Rinehart patents [77,78], and then further developed by Jou *et al.* [79]. The compound could also be made by modification of didemnin A with a patent covering this process being published in 1995 [80]. Further information as to synthetic methodologies of aplidine and other related compounds can be found in the excellent publication from the Joullie group published in 2012 [81].

The mechanism of action of this agent is still not fully identified but involves multiple regulatory pathways as can be seen in the discussion in the review by the PharmaMar group in 2012 [82], but the compound does demonstrate activity in a variety of cancers. These include a recent report of activity in pediatric medulloblastomas, with demonstrable partial responses and disease stabilization but the small patient number did not allow an efficacy determination [83].

Currently, this agent is shown as being in one Phase III trial in the NIH clinical trials database and in five Phase II trials covering a variety of leukemias and liposarcomas. What is also of interest is the recent report that didemnin B has been produced by fermentation of a free-living marine-sourced microbe, with the complete genomic cluster identified and production of didemnin B confirmed by “MALDI-TOF interrogation” of the metabolites in real time [84]. To date, no report of aplidine from this source has been published but it is highly likely that a microbe is the producer of either aplidine or that there is a simple oxidation of didemnin B to produce aplidine, since the only difference is the presence of a carbonyl in the side chain of aplidine instead of a C–OH in the same position in didemnin B.

### 4.2. Kahalalide F (PM-92102, Phase II; Figure 3, **13**)

This compound, a depsipeptide originally isolated from the sacoglossan mollusk *Elysia rufescens* and then subsequently isolated from the alga *Bryopsis pennata* upon which it feeds, was also found in an Indian Ocean *Elysia* but from a different species [85]. There is a possibility that the material is actually produced by a commensal microbe on the alga but this has not yet been definitively proven. The compound was licensed to PharmaMar by the University of Hawaii at Manoa (Honolulu, HI, USA) and then PharmaMar developed synthetic methods to produce the compound in bulk using peptide chemistry techniques.

It entered clinical trials against prostate cancer, malignant melanoma and non-small cell lung cancer, with a mechanism of action that involved oncosis (ischemic cell death) [86]. At this moment in time, it appears that PharmaMar does not have any current clinical trials underway from inspection of the NIH database, but in the EU Clinical Trials Register, a Phase II trial on non-small cell lung cancer is still ongoing in Spain under the EudraCT number 2004-001253-39. The compound was “out-licensed” for the treatment of psoriasis (a proliferative disease) to Medimetriks Pharmaceuticals (Fairfield, NJ, USA) in 2009 for other than oncology and neurology indications.

The original discoverer of this group of compounds published an interesting paper on selected kahalalide F analogs with antitumor and antifungal activities in 2011 [87], and recently in the middle of 2012, PharmaMar stopped development of the kahalalide derivative 1-[*N*-[(4*S*)-4-methyl-1-oxohexyl]-d-valine]-kahalalide F which they were developing as an antitumor agent under the generic name elisidepsin and trade name of Irvalec^®^ [82], even though it had activity in gastroesophageal tumors. This appeared to be a business decision due to the very low numbers of potential patients.

### 4.3. Plinabulin (NPI-2358, Phase II; Figure 3, **14**)

This compound, a simple modification of the terrestrial and also marine fungal metabolite halimide [88], entered Phase II clinical trials under Nereus Pharmaceuticals (San Diego, CA, USA). Two completed trials under that sponsor are shown in the NIH database, one at Phase I and the other at Phase I/II. However, no work/reports have appeared recently and with reports that Nereus Pharmaceuticals is no longer operative the fate of this compound is uncertain [89].

### 4.4. Marizomib^®^ (Salinosporamide A; NPI-0052; Phase I, Figure 3, **15**)

The story of this compound from its discovery from the marine actinomycete, *Salinispora tropica* and its identification as a proteasome inhibitor has been covered extensively in the scientific literature. The reports include work-up to give cGMP product from the first marine-medium based large-scale fermentation, through synthesis by a variety of chemists both in academia and companies, and identification of the biosynthetic cluster in the producing organism(s) [90,91,92,93,94].

Currently one clinical trial is shown in the NIH database as recruiting patients for a study in multiple myeloma (NCT00461045). What is of note is that the site now lists the sponsor as Triphase Research and Development Corporation, a CRO rather than Nereus Pharmaceuticals, and the other three completed Phase I trials in the NIH database no longer show Nereus but TriPhase. This may well confirm information about the current status of Nereus Pharmaceuticals [89].

### 4.5. PM-060184 (Phase I; Figure 3, **16**)

Recently, workers at PharmaMar reported the isolation and then total synthesis of two novel polyketides from the Madagascan sponge *Lithoplocamia lithistoides* [95]. These two novel agents PM050489 and PM060184 demonstrated sub-nanomolar *in vitro* activity in human cancer cell lines, also potent antimitotic activity, and specifically, demonstrated a new biochemical mechanism when interacting with tubulin [96]. The compounds also demonstrated potent *in vivo* activity in different animal models and one of the two, PM-060184 is currently in Phase I clinical trials (NCT01299636). Thus, even today, more than 25 years after the identification of the MOA of Taxol^®^ novel tubulin interaction mechanisms are still being discovered.

## 5. Conclusions

In an entirely different aspect of marine pharmacology, the work described in the first section of this review, with “toxins” controlling cancer-related pain, is a beautiful example of where agents considered to be deadly poisons to humans, tetrodotoxin and the *Conus* peptides, are now leading to potential drugs for use in humans.

In a considerable number of papers covering the topic of natural product-based antitumor drugs (irrespective of the natural source), compounds are quoted as being in clinical trials, even though no new trial has been reported in the previous four-plus years, and earlier trials are listed as completed. From this perspective, there are two papers, one in this journal in 2010 [97], and a very recent and truly comprehensive compilation of clinical trials of a large number of marine-related drugs and drug candidates [98], that could be considered to have partially covered the topic from a marine source perspective. In these two papers, however, a significant number of the agents discussed are no longer in clinical trials.

In contrast to this approach, we have checked for current or recent antitumor trials in all of the compounds above (except for the three listed as preclinical) and have demonstrated that, although the number of marine-derived agents in active clinical studies en route to approval is small compared to earlier years, all listed have current or recent clinical trials shown in either the NIH or the corresponding EU clinical trials databases.

We used the qualifier “small” above even though there are 24 ADCs (5 ADCs at Phase II, 16 ADCs at Phase I and 3 ADCs in advanced preclinical status) that are using either auristatin E or auristatin F as their warheads. If one is being conservative, then these are composed of only two different basal structures with the other differences being in the monoclonal antibody and the method of linkage. We would also be remiss in not pointing out that none of these ADCs would have been made except for the discovery of the dolastatins and subsequent syntheses in the middle to late 1970s [99].

With the number of marine-sourced compounds in the literature now over 25,000, there are many other agents just “waiting in the wings” for their chance of stardom; it is our task as marine natural product chemists to find them and, ultimately, to develop them as either drugs or leads thereto. We would be remiss in not mentioning that nowadays it is recognized that the probable source of most of the agents that we have described are single celled organisms, ranging from eubacteria through to eukaryotes such as fungi and protists. As yet, we are not aware of any published compounds from the archaea that have antitumor activity, but they may well occur in due course.
